# Supplementary material for: Recombinant Simian Varicella Virus-Simian Immunodeficiency Virus Vaccine Induces T and B Cell Functions and Provides Partial Protection against Repeated Mucosal SIV Challenges in Rhesus Macaques
Source: Viruses. 2022 Dec 17;14(12):2819. doi: 10.3390/v14122819 (PMC9853323; doi:10.3390/v14122819)
Supplement: Supplementary file 1 [file viruses-14-02819-s001.zip › viruses-2045882-supplementary-revise/Supplemental Tables S1-S5_FINAL- revise.pdf]

**Table S1: Animal group distribution**

| Group | Animal Number | Age (Years) | Body Weight (Kg) |
|---|---|---|---|
| Vaccine | LK45 | 5.5 | 4.6 |
| | LK50 | 5.3 | 5.9 |
| | LK54 | 5.0 | 5.7 |
| | LK56 | 4.6 | 5.5 |
| | LK57 | 4.5 | 5.3 |
| | LK58 | 4.4 | 5.2 |
| | LK59 | 4.4 | 6.0 |
| | LK60 | 4.4 | 5.1 |
| Control | LK42 | 6.4 | 5.2 |
| | LK46 | 5.5 | 5.3 |
| | LK51 | 5.2 | 5.9 |
| | LK53 | 5.1 | 5.2 |
| | LK55 | 4.6 | 6.0 |
| | LP32 | 4.6 | 5.0 |
| | LP33 | 5.5 | 5.0 |

All macaques were MHC typed and negative for Mamu-A*01, B*08 and B*17 alleles.

**Table S2: List of monoclonal antibodies used for T-cell phenotyping and antigen specific cytokine flow cytometry assays**

| Antibody | Clone | Amount used (µL) | Source |
|---|---|---|---|
| Anti-human CD3 | SP34-2 | 5 | BD Biosciences, CA |
| Anti-human CD4 | L200 | 3 | BD Biosciences, CA |
| Anti-human CD8 | SK1 | 3 | BD Biosciences, CA |
| Anti-human CD25 | M-A251 | 5 | Biolegend, CA |
| Anti-human CD28 | CD28.2 | 5 | BD Biosciences, CA |
| Anti-human CD38 | OKT10 | 5 | NHP Reagent Resource Program, NIH |
| Anti-NHP CD45 | D058-1283 | 10 | BD Biosciences, CA |
| Anti-human CD69 | FN50 | 5 | Biolegend, CA |
| Anti-human CD95 | DX2 | 5 | BD Biosciences, CA |
| Anti-human CD107a | H4A3 | 20 | BD Biosciences, CA |
| Anti-human CD107b | H4B4 | 5 | BD Biosciences, CA |
| Anti-human HLA-DR | G46-6 | 5 | BD Biosciences, CA |
| Anti-human IFNγ | B27 | 3 | Biolegend, CA |
| Anti-human IL-2 | JES3-9D7 | 5 | Biolegend, CA |
| Anti-human IL-4 | 8D4-8 | 5 | BD Biosciences, CA |
| Anti-human IL-5 | JES1-39D10 | 5 | Sony Biotech, CA |
| Anti-human IL-21 | 3A3-N2 | 5 | Sony Biotech, CA |
| Anti-mouse Ki67 | B56 | 20 | BD Biosciences, CA |
| Anti-human TNFα | Mab11 | 5 | Biolegend, CA |

**Table S3**: **Statistical Analysis of different cell types and tissues in vaccine Group**

| Cell Types/Tissue | Vaccine timepoint Comparison | Adjusted p value | Mean values ± Standard Errors (%) |
|---|---|---|---|
| CM CD4+/ PBMC | pV#2 vs. pV#3 | 0.0007 | 40.9 ± 2.2 vs. 56.8 ± 3.8 |
| Naïve CD4+/ LN | pV#2 vs. pV#5 | 0.025 | 71.0 ± 3.9 vs. 53.6 ± 5.4 |
| CM CD4+/ LN | Pre vs. pV#5 | 0.045 | 29.1 ± 3.7 vs. 46.0 ± 6.0 |
| | pV#2 vs. pV#5 | 0.003 | 22.8 ± 3.3 vs. 46.0 ± 6.0 |
| EM CD4+/ LN | Pre vs. pV#2 | 0.009 | 6.3 ± 0.8 vs. 3.0 ± 0.4 |
| | Pre vs. pV#3 | 0.017 | 6.3 ± 0.8 vs. 3.2 ± 0.7 |
| | Pre vs. pV#5 | <0.0001 | 6.3 ± 0.8 vs. 1.2 ± 0.5 |
| | pV#2 vs. pV#4 | 0.009 | 3.0 ± 0.4 vs. 6.3 ± 0.9 |
| | pV#3 vs. pV#4 | 0.017 | 3.2 ± 0.7 vs. 6.3 ± 0.9 |
| | pV#4 vs. pV#5 | <0.0001 | 6.3 ± 0.9 vs. 1.2 ± 0.5 |
| CD4+/Jej LPL | Pre vs. SIV+ | 0.020 | 47.3 ± 4.5 vs. 25.4 ± 4.1 |
| | pV#2 vs. SIV+ | 0.012 | 53.7 ± 7.9 vs. 25.4 ± 4.1 |
| | pV#3 vs. SIV+ | <0.0001 | 72.4 ± 2.8 vs. 25.4 ± 4.1 |
| | pV#5 vs. SIV+ | 0.0004 | 56.3 ± 6.1 vs. 25.4 ± 4.1 |
| CD4+/Rectal LPL | Pre vs. SIV+ | <0.0001 | 62.9 ± 3.6 vs. 23.7 ± 4.5 |
| | pV#2 vs. SIV+ | 0.0004 | 53.7 ± 7.9 vs. 23.7 ± 4.5 |
| | pV#3 vs. SIV+ | <0.0001 | 69.9 ± 2.5 vs. 23.7 ± 4.5 |
| | pV#4 vs. SIV+ | <0.0001 | 54.3 ± 2.8 vs. 23.7 ± 4.5 |
| | pV#5 vs. SIV+ | <0.0001 | 57.7 ± 3.6 vs. 23.7 ± 4.5 |
| CM CD4+/Jej LPL | Pre vs. pV#2 | 0.002 | 61.4 ± 6.7 vs. 94.3 ± 0.9 |
| | Pre vs. pV#3 | 0.029 | 61.4 ± 6.7 vs. 81.6 ± 3.5 |
| | pV#2 vs. pV#5 | 0.010 | 94.3 ± 0.9 vs. 66.1 ± 3.9 |
| EM CD4+/Jej LPL | Pre vs. pV#2 | 0.001 | 35.8 ± 6.9 vs. 1.5 ± 0.5 |
| | Pre vs. pV#3 | 0.026 | 35.8 ± 6.9 vs. 15.5 ± 3.0 |
| | pV#2 vs. pV#5 | 0.015 | 1.5 ± 0.5 vs. 28.3 ± 3.9 |
| CM CD4+/Rectal LPL | Pre vs. pV#2 | 0.0003 | 76.1 ± 1.5 vs. 94.3 ± 0.9 |
| | Pre vs. pV#3 | 0.011 | 76.1 ± 1.5 vs. 86.9 ± 3.8 |
| | Pre vs. pV#5 | 0.020 | 76.1 ± 1.5 vs. 86.2 ± 1.9 |
| CM CD8+/Jej LPL | Pre vs. pV#2 | <0.0001 | 61.3 ± 2.3 vs. 94.3 ± 0.6 |
| | Pre vs. pV#3 | <0.0001 | 61.3 ± 2.3 vs. 89.4 ± 3.3 |
| | Pre vs. pV#5 | <0.0001 | 61.3 ± 2.3 vs. 30.6 ± 5.5 |

| | | | |
|---|---|---|---|
| | pV#3 vs. pV#4 | <0.0001 | 89.4 ± 3.3 vs. 54.1 ± 2.4 |
| | pV#3 vs. pV#5 | <0.0001 | 89.4 ± 3.3 vs. 30.6 ± 5.5 |
| | pV#4 vs. pV#5 | 0.0003 | 54.1 ± 2.4 vs. 30.6 ± 5.5 |
| EM CD8+/Jej LPL | Pre vs. pV#2 | <0.0001 | 36.2 ± 2.8 vs. 3.5 ± 0.5 |
| | Pre vs. pV#3 | 0.0001 | 36.2 ± 2.8 vs. 9.6 ± 3.2 |
| | Pre vs. pV#5 | <0.0001 | 36.2 ± 2.8 vs. 67.7 ± 5.9 |
| | pV#3 vs. pV#4 | <0.0001 | 9.6 ± 3.2 vs. 43.5 ± 2.2 |
| | pV#3 vs. pV#5 | <0.0001 | 9.6 ± 3.2 vs. 67.7 ± 5.9 |
| | pV#4 vs. pV#5 | 0.0004 | 43.5 ± 2.2 vs. 67.7 ± 5.9 |
| CM CD8+/Rectal LPL | Pre vs. pV#2 | 0.0001 | 57.9 ± 3.5 vs. 94.7 ± 0.5 |
| | Pre vs. pV#3 | 0.003 | 57.9 ± 3.5 vs. 81.4 ± 5.2 |
| | pV#3 vs. pV#4 | 0.010 | 81.4 ± 5.2 vs. 60.6 ± 5.1 |
| | pV#3 vs. pV#5 | <0.0001 | 81.4 ± 5.2 vs. 42.6 ± 3.2 |
| | pV#4 vs. pV#5 | 0.034 | 60.6 ± 5.1 vs. 42.6 ± 3.2 |
| EM CD8+/Rectal LPL | Pre vs. pV#2 | 0.0002 | 39.0 ± 3.6 vs. 2.8 ± 0.6 |
| | Pre vs. pV#3 | 0.007 | 39.0 ± 3.6 vs. 17.1 ± 5.2 |
| | pV#3 vs. pV#4 | 0.018 | 17.1 ± 5.2 vs. 36.8 ± 5.1 |
| | pV#3 vs. pV#5 | <0.0001 | 17.1 ± 5.2 vs. 55.3 ± 3.4 |
| | pV#4 vs. pV#5 | 0.029 | 36.8 ± 5.1 vs. 55.3 ± 3.4 |
| CD4+CD38+/Jej LPL | Pre vs. pV#2 | 0.005 | 32.6 ± 4.1 vs. 11.9 ± 1.9 |
| | Pre vs. pV#3 | <0.0001 | 32.6 ± 4.1 vs. 2.3 ± 0.9 |
| | Pre vs. pV#5 | 0.015 | 32.6 ± 4.1 vs. 17.4 ± 2.9 |
| | pV#3 vs. pV#4 | 0.007 | 2.3 ± 0.9 vs. 19.5 ± 5.1 |
| | pV#4 vs. pV#5 | 0.012 | 19.5 ± 5.1 vs. 17.4 ± 2.9 |
| CD4+CD38+/Rectal LPL | pV#3 vs. pV#4 | 0.019 | 1.1± 0.5 vs. 13.1 ± 3.9 |
| | pV#3 vs. pV#5 | 0.019 | 1.1± 0.5 vs. 12.7 ± 2.4 |
| CD8+CD38+/Jej LPL | Pre vs. pV#3 | <0.0001 | 19.6 ± 2.4 vs. 1.4 ± 0.5 |
| | pV#2 vs. pV#3 | 0.035 | 13.3 ± 3.4 vs. 1.4 ± 0.5 |
| | pV#3 vs. pV#4 | <0.0001 | 1.4 ± 0.5 vs. 21.4 ± 3.6 |
| | pV#3 vs. pV#5 | 0.003 | 1.4 ± 0.5 vs. 14.2 ± 2.4 |
| CD8+CD38+/Rectal LPL | Pre vs. pV#3 | 0.009 | 16.4 ± 3.8 vs. 1.4 ± 0.7 |
| CD4+HLADR+/Jej LPL | Pre vs. pV#3 | 0.003 | 9.1 ± 0.6 vs. 3.6 ± 0.5 |
| CD8+HLADR+/Rectal LPL | Pre vs. pV#4 | 0.024 | 5.2 ± 2.4 vs. 12.8 ± 1.3 |
| CD4+CD69+/Jej LPL | Pre vs. pV#2 | <0.0001 | 64.8 ± 4.3 vs. 2.7 ± 0.9 |
| | Pre vs. pV#3 | <0.0001 | 64.8 ± 4.3 vs. 30.6 ± 4.1 |

| | | | |
|---|---|---|---|
| | Pre vs. pV#5 | <0.0001 | 64.8 ± 4.3 vs. 31.8 ± 1.8 |
| CD4+CD69+/Rectal LPL | Pre vs. pV#2 | <0.0001 | 43.8 ± 7.2 vs. 3.0 ± 2.0 |
| | Pre vs. pV#3 | 0.0007 | 43.8 ± 7.2 vs. 16.6 ± 2.9 |
| | Pre vs. pV#5 | 0.006 | 43.8 ± 7.2 vs. 22.4 ± 1.1 |
| CD8+CD69+/Jej LPL | Pre vs. pV#2 | <0.0001 | 64.2 ± 4.2 vs. 7.8 ± 1.6 |
| | Pre vs. pV#3 | <0.0001 | 64.2 ± 4.2 vs. 36.7 ± 4.7 |
| | Pre vs. pV#5 | 0.013 | 64.2 ± 4.2 vs. 46.4 ± 2.8 |

## Table S4: SIV-antigen specific cytokine responses in lymph node tissue during pV#3 and pV#4 vaccination phase

| Macaque | Antigen# | T cells | pV#3 | | | | | | | pV#4 | | | | | | |
|---|---|---|---|---|---|---|---|---|---|---|---|---|---|---|---|---|
| | | | CD107a/b | IFNγ | IL2 | IL4 | IL5 | IL21 | TNFα | CD107a/b | IFNγ | IL2 | IL4 | IL5 | IL21 | TNFα |
| LK50 | Gag | CD4 | | | 0.10 | 0.22 | | | | | | | | | | |
| LK57 | Gag | CD4 | | 0.26 | | | | | | | | | 3.66 | | | 0.11 |
| LK58 | Gag | CD4 | | | | | | | 0.24 | | | | | | | |
| LK50 | Gag | CD8 | 0.34 | | | | 0.24 | | | | | | | | | |
| LK57 | Gag | CD8 | | | | | | | | 0.74 | 0.24 | | 4.11 | 0.10 | 0.09 | |
| LK58 | Gag | CD8 | | | 0.26 | 0.52 | | | | | | | 0.61 | | | 0.24 |
| LK45 | Gag | CD4 | | | | | | | | | | | 1.23 | | | |
| LK54 | Gag | CD4 | | | | | | | | | 0.24 | | 0.63 | 0.24 | | |
| LK56 | Gag | CD4 | 0.24 | 0.54 | 0.32 | | 0.38 | 0.34 | | | | | 1.25 | | | |
| LK59 | Gag | CD4 | | | | | | | | | | | | | | 0.20 |
| LK60 | Gag | CD4 | | | | 0.13 | | | | | | | | | | |
| LK45 | Gag | CD8 | | | | | | | | | 0.10 | | 2.46 | | | |
| LK54 | Gag | CD8 | | | | | | | | 0.15 | 0.36 | | | 0.34 | 0.43 | 0.33 |
| LK56 | Gag | CD8 | 0.22 | 0.22 | 0.22 | | | | 2.04 | | | | 1.86 | | | |
| LK59 | Gag | CD8 | | | | | | | | | 0.09 | | 1.58 | 0.14 | | |
| LK60 | Gag | CD8 | | | 0.31 | 0.10 | | | | | | | | | | |
| LK50 | Env | CD4 | | | 0.13 | 0.45 | | | | | | | | 0.24 | | |
| LK57 | Env | CD4 | | 0.84 | 0.58 | | | | | | | | 1.85 | | | |
| LK58 | Env | CD4 | 1.83 | 0.21 | | | | | | | | | | | | 0.31 |
| LK50 | Env | CD8 | | | | 0.45 | | 0.65 | | | | | | 0.21 | | |
| LK57 | Env | CD8 | | 1.01 | 0.33 | | | | | 0.71 | | | | 0.14 | 0.08 | |
| LK58 | Env | CD8 | 2.48 | 0.39 | 0.46 | 1.58 | | 1.17 | | | | 0.28 | 0.93 | 0.82 | 1.03 | 1.36 |
| LK45 | Env | CD4 | | | | | | 3.36 | | | | | | | | |
| LK54 | Env | CD4 | | 1.79 | 0.23 | 0.64 | | | | | 0.19 | | | 0.26 | | |
| LK56 | Env | CD4 | 0.31 | 0.10 | 0.77 | 0.46 | 0.77 | 5.43 | 1.82 | | | | 0.13 | | | |
| LK59 | Env | CD4 | | | | | | | | | | | | | | 0.11 |
| LK60 | Env | CD4 | | | | 0.39 | | 0.61 | | | | | | | | |
| LK45 | Env | CD8 | | | | | | | | | | | 3.12 | | | |
| LK54 | Env | CD8 | 0.64 | 0.64 | 0.21 | | 0.37 | | | | 0.29 | | 0.71 | 0.22 | 0.29 | 0.16 |
| LK56 | Env | CD8 | 1.48 | 1.21 | 1.21 | 0.93 | 1.15 | 8.00 | 2.29 | 0.54 | 0.22 | | 1.69 | | 0.07 | |
| LK59 | Env | CD8 | | | | | 1.49 | | | | 0.14 | 0.09 | 1.34 | | 0.14 | |
| LK60 | Env | CD8 | | | 0.18 | 0.17 | | | | | | | | | | |

#Peptide specific responses are shown as percentage for CD3+CD4+ and CD3+CD8+ T cell subsets. Although both Gag and Env peptides pools were tested, only positive responses are shown.

## Table S5: SIV-antigen specific cytokine responses in PBMC during pV#3 and pV#4 vaccination phase

| Macaque | Antigen# | T cells | pV#3 CD107a/b | pV#3 IFNγ | pV#3 IL2 | pV#3 IL4 | pV#3 IL5 | pV#3 IL21 | pV#3 TNFα | pV#4 CD107a/b | pV#4 IFNγ | pV#4 IL2 | pV#4 IL4 | pV#4 IL5 | pV#4 IL21 | pV#4 TNFα |
|---|---|---|---|---|---|---|---|---|---|---|---|---|---|---|---|---|
| LK50 | Gag | CD4 | | | | | | | | 0.34 | | | | | 0.23 | |
| LK57 | Gag | CD4 | | | | | | | | 0.54 | | | 3.73 | | | |
| LK58 | Gag | CD4 | 0.34 | 0.16 | | 2.63 | | 0.96 | 0.21 | 0.58 | | | | | | |
| LK50 | Gag | CD8 | | | | | | | | | | | | | | |
| LK57 | Gag | CD8 | | | | 0.24 | | | | | | | 4.74 | | | |
| LK58 | Gag | CD8 | 2.57 | 0.23 | | 0.15 | 0.15 | 0.89 | 1.21 | 0.90 | | | | | | |
| LK45 | Gag | CD4 | | 0.08 | | | | | 0.05 | | | | 1.72 | | | |
| LK54 | Gag | CD4 | | | | 2.69 | | 1.16 | 0.08 | 0.61 | | | | | | |
| LK56 | Gag | CD4 | | | | 0.12 | | | | 0.10 | | | | | | |
| LK59 | Gag | CD4 | | | | | | | | | 0.08 | 0.06 | 1.43 | | | 0.05 |
| LK60 | Gag | CD4 | | | | | | | | 0.35 | | | | | 0.14 | 0.23 |
| LK45 | Gag | CD8 | | | | | | 1.62 | | | | | 3.62 | | | |
| LK54 | Gag | CD8 | | | | 0.19 | | 0.98 | 0.37 | 0.39 | | | | 0.05 | | |
| LK56 | Gag | CD8 | | | | | | | | | | | | | | |
| LK59 | Gag | CD8 | | | | | 0.25 | | | | | | 3.78 | 0.36 | | |
| LK60 | Gag | CD8 | | | | | | | | 0.14 | | | | | | |
| LK50 | Env | CD4 | | | | | | | | 0.33 | | | | | | |
| LK57 | Env | CD4 | | | | | | | | 0.23 | | | 1.98 | | | |
| LK58 | Env | CD4 | 0.64 | | | | | 2.44 | 0.20 | 1.46 | | | | | | |
| LK50 | Env | CD8 | | | | | | | | | | | | | | |
| LK57 | Env | CD8 | | | | | | | | | | | 4.39 | | | |
| LK58 | Env | CD8 | | | | 0.17 | 0.09 | 1.58 | 0.84 | 1.28 | | | | | | |
| LK45 | Env | CD4 | | 0.27 | 0.08 | 0.13 | | | | 0.16 | | | 0.95 | | 0.08 | |
| LK54 | Env | CD4 | | | | | | | | 1.82 | | | | 0.13 | 0.27 | |
| LK56 | Env | CD4 | | 0.24 | 0.07 | 0.26 | 0.33 | 1.97 | | | | | | | | |
| LK59 | Env | CD4 | | | | | | | | | | | 1.3 | | | |
| LK60 | Env | CD4 | | | | | | | | 2.34 | | | | | 0.96 | 0.81 |
| LK45 | Env | CD8 | | | 0.21 | | 0.66 | | | | | | 2.93 | | 0.26 | |
| LK54 | Env | CD8 | | 0.07 | 0.13 | 0.16 | | 1.50 | 0.24 | 3.00 | | | | 0.11 | 0.81 | |
| LK56 | Env | CD8 | | 0.11 | | | 0.34 | 2.08 | 1.27 | | | | | | 0.05 | |
| LK59 | Env | CD8 | | | | | | | | | | | 4.35 | | 0.08 | |
| LK60 | Env | CD8 | | | | | | | | 1.04 | | | | | | |

#Peptide specific responses are shown as percentage for CD3+CD4+ and CD3+CD8+ T cell subsets. Although both Gag and Env peptides pools were tested, only positive responses are shown.
