# Supplementary material for: Recombinant Simian Varicella Virus-Simian Immunodeficiency Virus Vaccine Induces T and B Cell Functions and Provides Partial Protection against Repeated Mucosal SIV Challenges in Rhesus Macaques
Source: Viruses. 2022 Dec 17;14(12):2819. doi: 10.3390/v14122819 (PMC9853323; doi:10.3390/v14122819)
Supplement: Supplementary file 1 [file viruses-14-02819-s001.zip › viruses-2045882-supplementary-revise/Supplementary Figures S1-S8_FINAL.pdf]

Figure S1

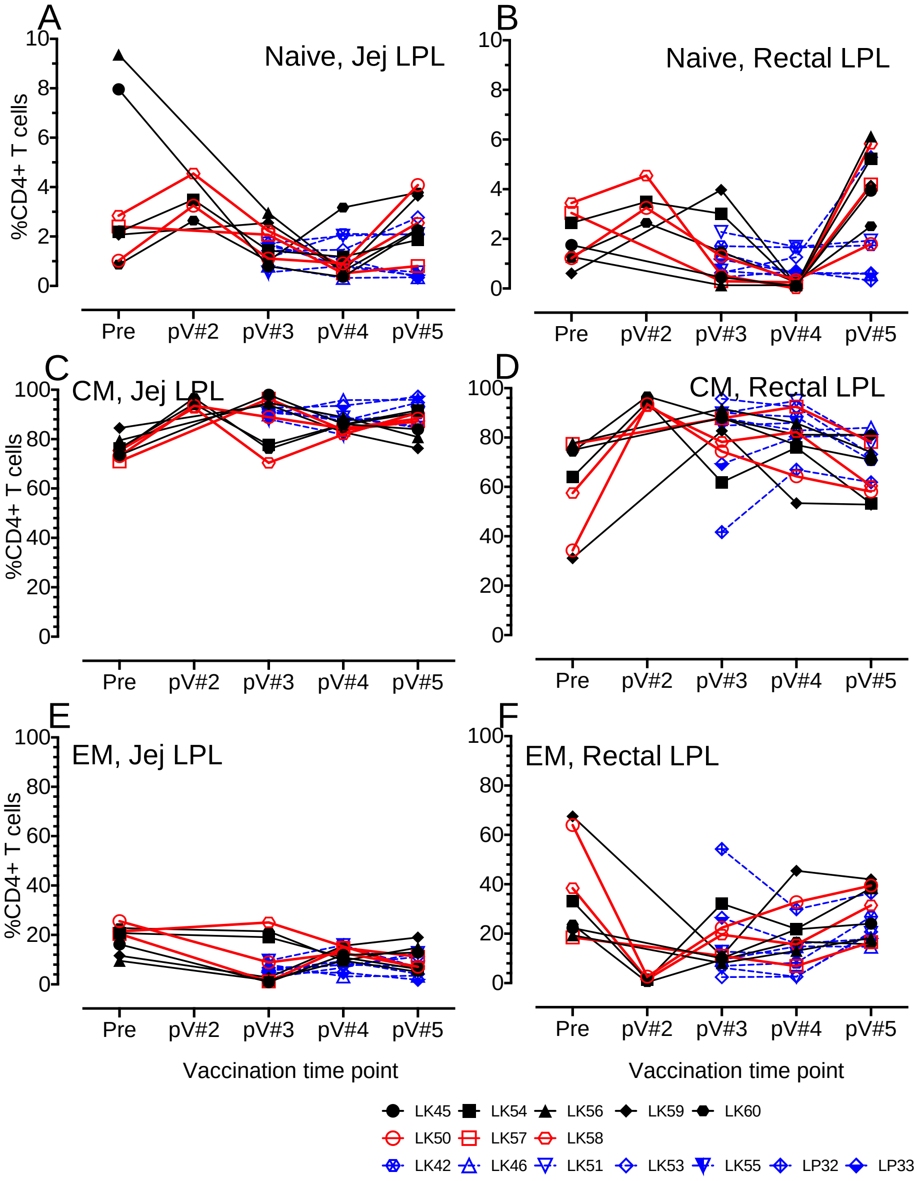
A
Naive, Jej LPL
B
Naive, Rectal LPL
C
CM, Jej LPL
D
CM, Rectal LPL
E
EM, Jej LPL
F
EM, Rectal LPL
%CD4+ T cells
Pre
pV#2
pV#3
pV#4
pV#5
Vaccination time point
LK45
LK54
LK56
LK59
LK60
LK50
LK57
LK58
LK42
LK46
LK51
LK53
LK55
LP32
LP33

Figure S2

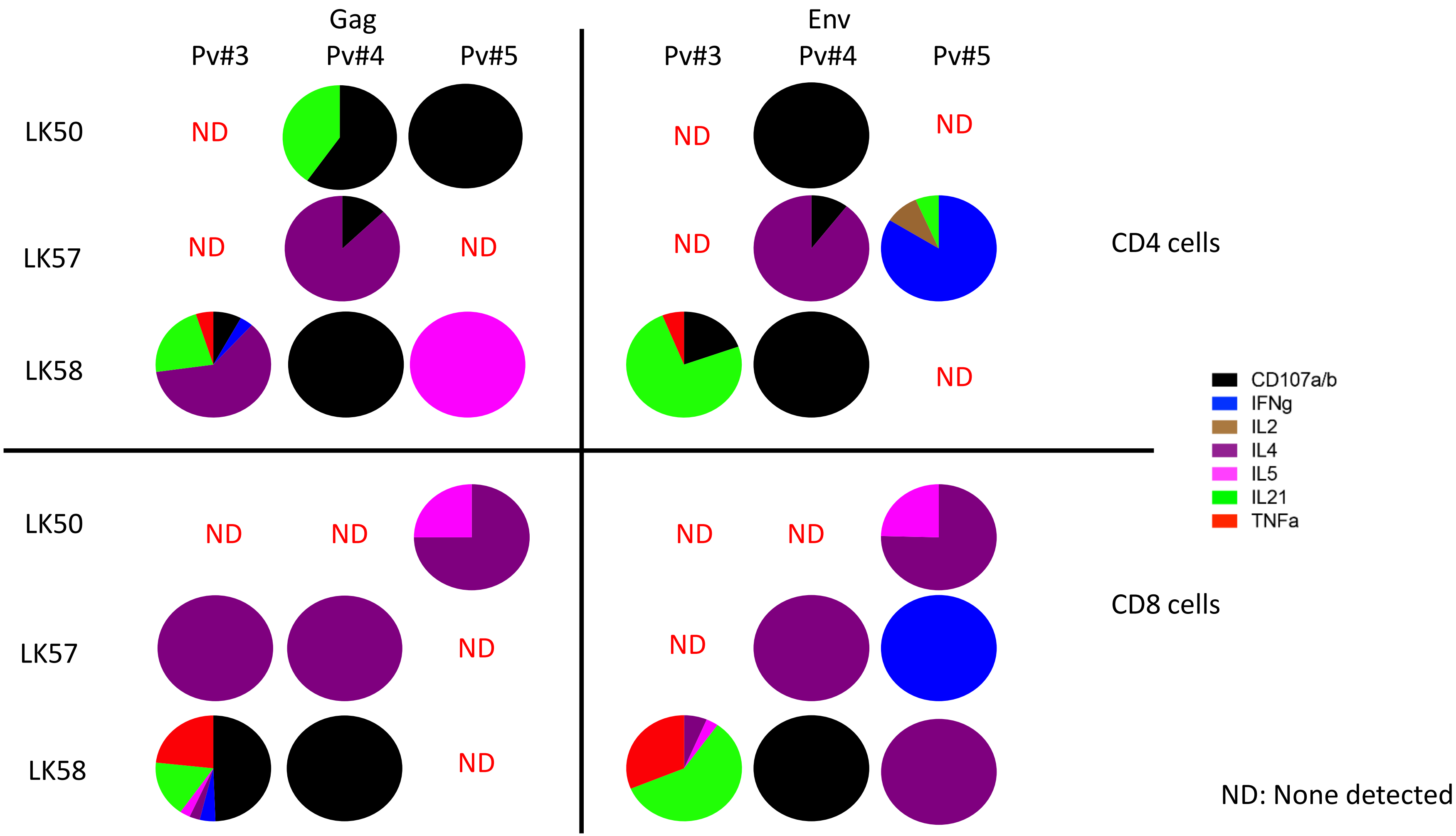
Gag
Env
Pv#3
Pv#4
Pv#5
Pv#3
Pv#4
Pv#5
LK50
LK57
LK58
LK50
LK57
LK58
ND
ND
ND
ND
ND
ND
ND
ND
ND
ND
ND
ND
ND
ND
ND
CD4 cells
CD8 cells
CD107a/b
IFNg
IL2
IL4
IL5
IL21
TNFa
ND: None detected

Figure S3

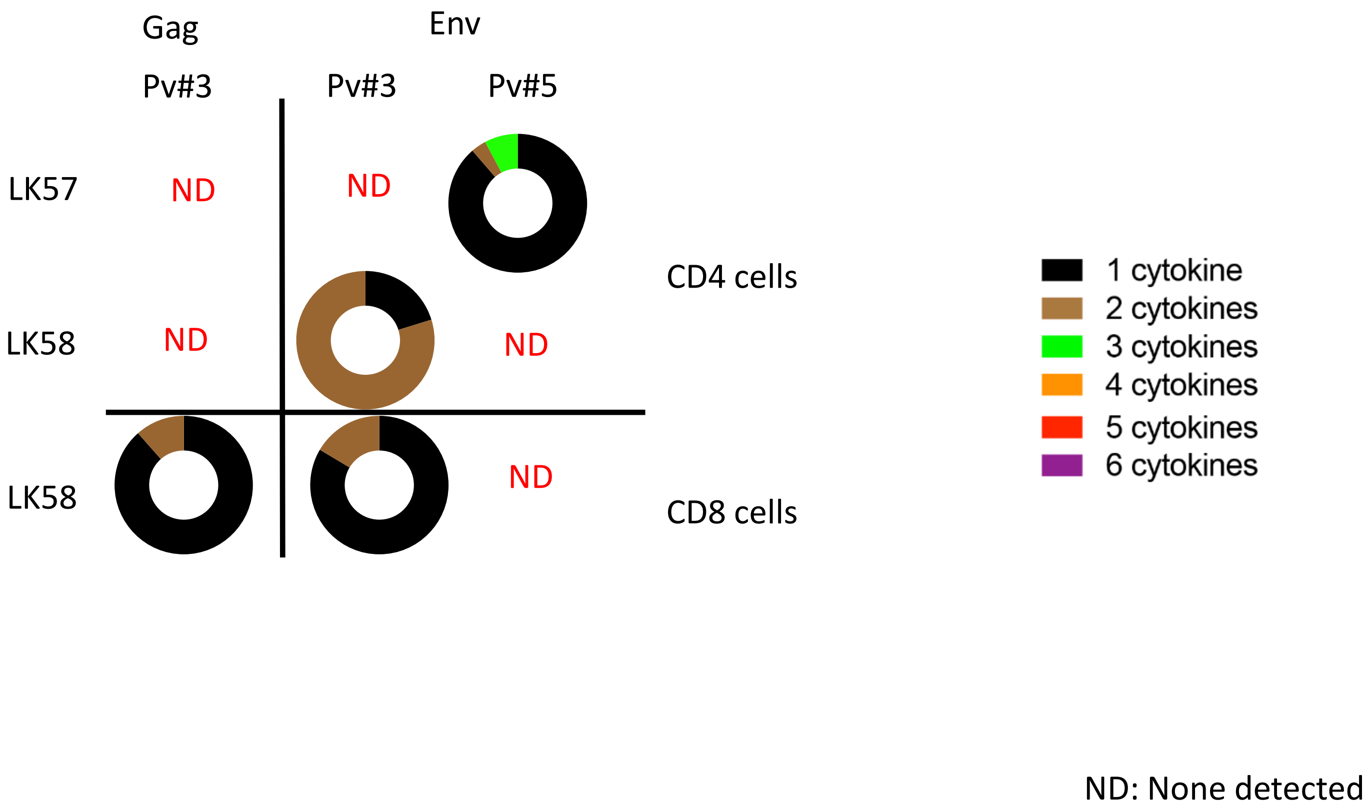
Gag
Env
Pv#3
Pv#3
Pv#5
LK57
ND
ND
LK58
ND
ND
LK58
ND
CD4 cells
CD8 cells
1 cytokine
2 cytokines
3 cytokines
4 cytokines
5 cytokines
6 cytokines
ND: None detected

Figure S4

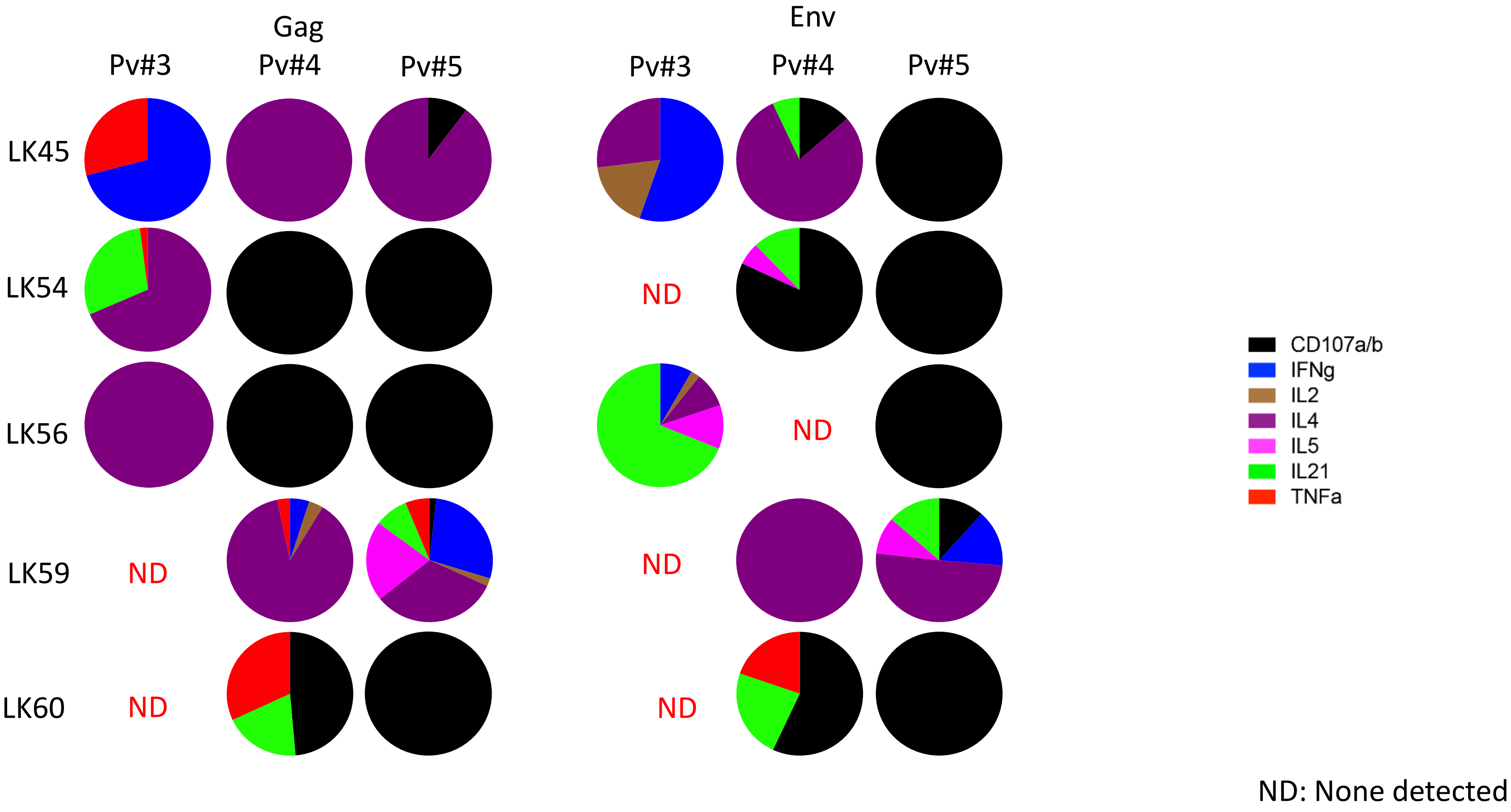
Gag
Env
Pv#3
Pv#4
Pv#5
Pv#3
Pv#4
Pv#5
LK45
LK54
LK56
LK59
LK60
ND
ND
ND
ND
ND
ND
ND
CD107a/b
IFNg
IL2
IL4
IL5
IL21
TNFa
ND: None detected

Figure S5

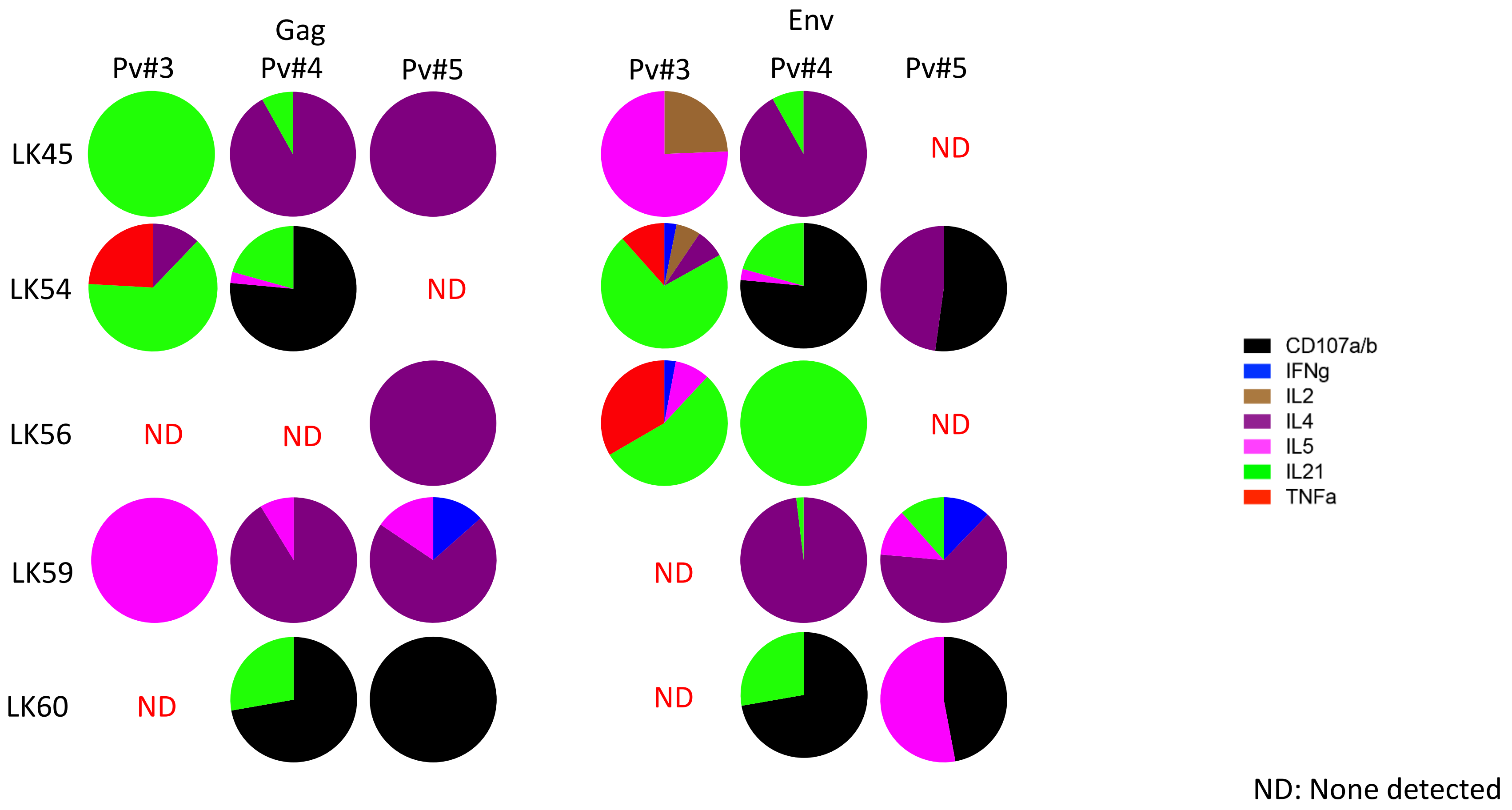
Gag
Env
Pv#3
Pv#4
Pv#5
Pv#3
Pv#4
Pv#5
LK45
LK54
LK56
LK59
LK60
ND
ND
ND
ND
ND
ND
ND
ND
CD107a/b
IFNg
IL2
IL4
IL5
IL21
TNFa
ND: None detected

Figure S6

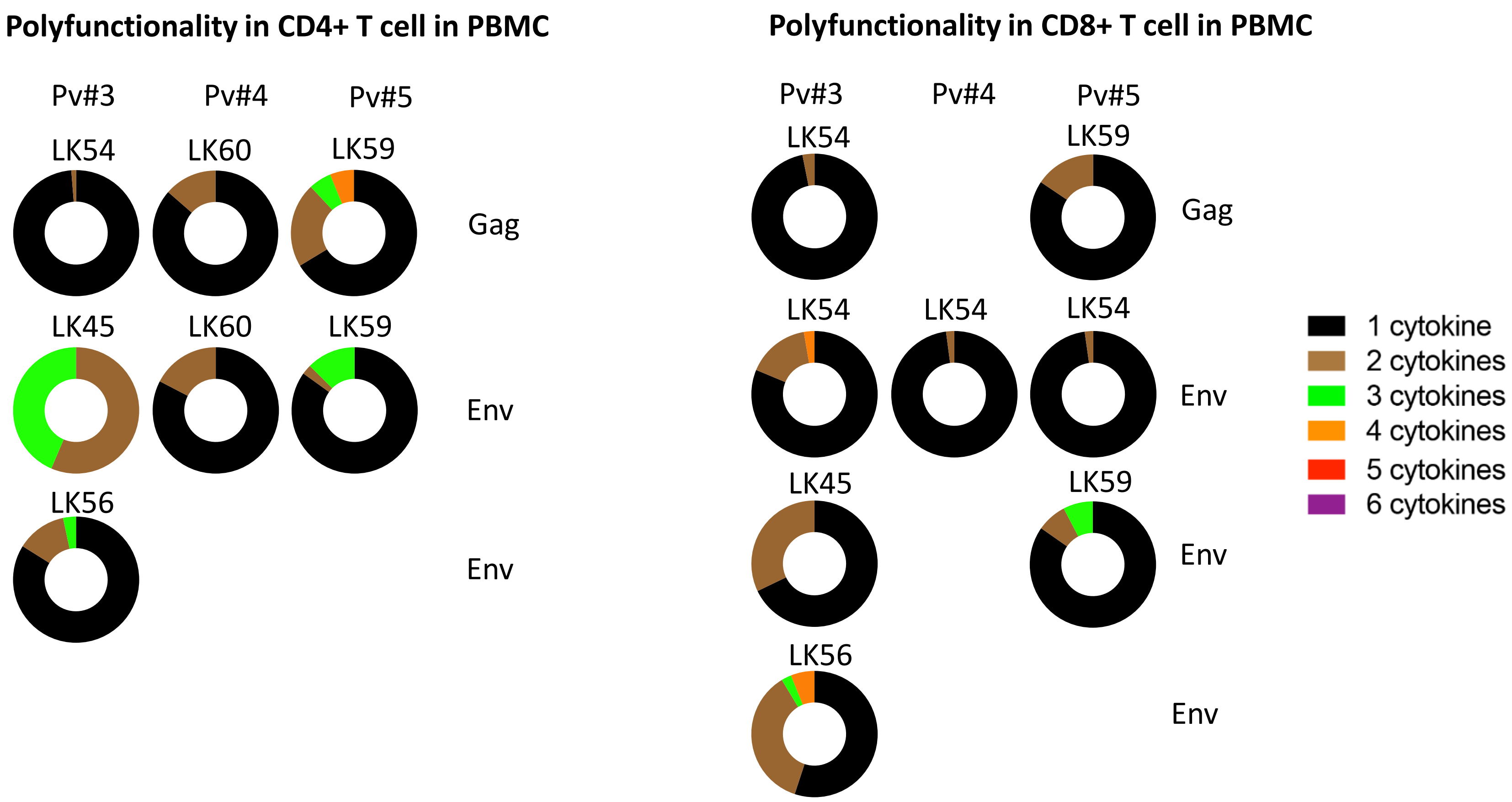
Polyfunctionality in CD4+ T cell in PBMC
Pv#3
Pv#4
Pv#5
LK54
LK60
LK59
Gag
LK45
LK60
LK59
Env
LK56
Env
Polyfunctionality in CD8+ T cell in PBMC
Pv#3
Pv#4
Pv#5
LK54
LK59
Gag
LK54
LK54
LK54
Env
LK45
LK59
Env
LK56
Env
1 cytokine
2 cytokines
3 cytokines
4 cytokines
5 cytokines
6 cytokines

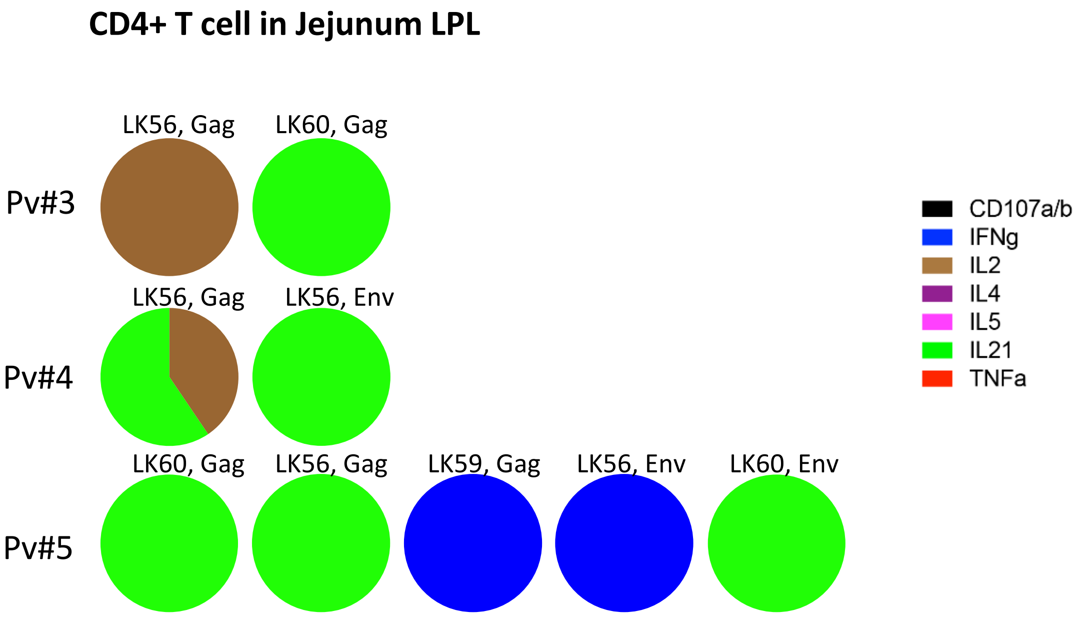

Note: Assays performed for the following jejunum LPL samples and antigen

PV#3: LK50 (Gag), LK54 (Gag), LK58 (Gag), LK60 (Gag), LK45 (Gag), LK56 (Gag), LK57 (Gag), LK53 (Gag), LK55 (Gag)

PV#4: LK56 (Gag & Env), LK57 (Env), LK58 (Gag & Env), LK59 (Gag)

PV#5: LK46 (Gag), LK45 (Gag), LK56 (Gag & Env), LK57 (Gag), LK59 (Gag), LK60 (Gag & Env), LP32 (Gag)

Figure S8

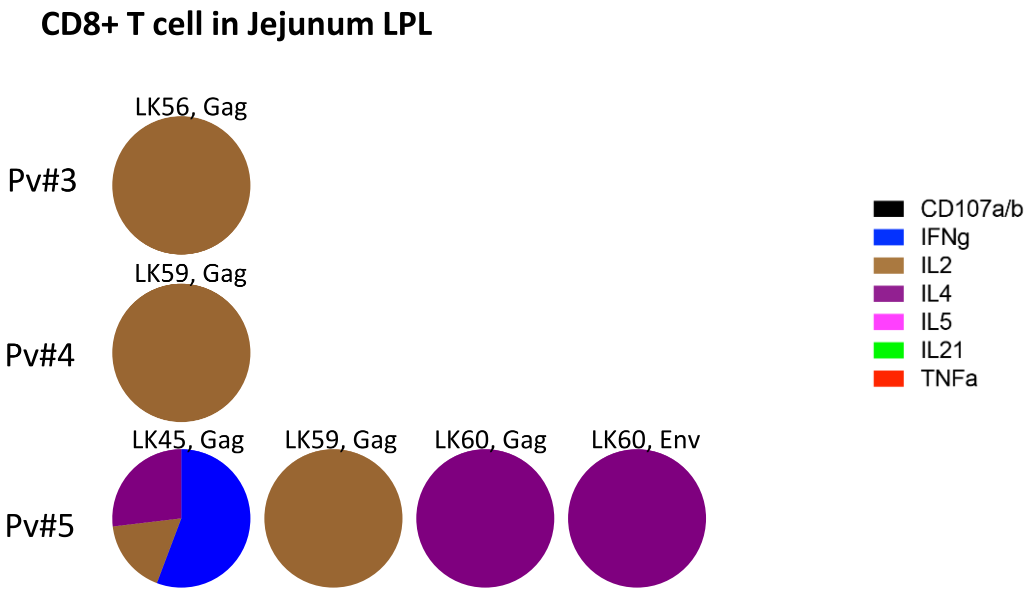

Note: Assays performed for the following jejunum LPL samples and antigen

PV#3: LK50 (Gag), LK54 (Gag), LK58 (Gag), LK60 (Gag), LK45 (Gag), LK56 (Gag), LK57 (Gag), LK53 (Gag), LK55 (Gag)

PV#4: LK56 (Gag & Env), LK57 (Env), LK58 (Gag & Env), LK59 (Gag)

PV#5: LK46 (Gag), LK45 (Gag), LK56 (Gag & Env), LK57 (Gag), LK59 (Gag), LK60 (Gag & Env), LP32 (Gag)
